# Adapting Genotyping-by-Sequencing for Rice F2 Populations

**DOI:** 10.1534/g3.116.038190

**Published:** 2017-01-11

**Authors:** Tomoyuki Furuta, Motoyuki Ashikari, Kshirod K. Jena, Kazuyuki Doi, Stefan Reuscher

**Affiliations:** * Bioscience and Biotechnology Center, Nagoya University, 464-8601, Japan; † Plant Breeding Division, International Rice Research Institute, 1301 Manila, Philippines; ‡ Associated Field Science and Research Center, Nagoya University, 470-0151, Japan

**Keywords:** genotyping-by-sequencing, SNP marker, rice breeding, trait mapping

## Abstract

Rapid and cost-effective genotyping of large mapping populations can be achieved by sequencing a reduced representation of the genome of every individual in a given population, and using that information to generate genetic markers. A customized genotyping-by-sequencing (GBS) pipeline was developed to genotype a rice F2 population from a cross of *Oryza sativa* ssp. *japonica* cv. Nipponbare and the African wild rice species *O. longistaminata*. While most GBS pipelines aim to analyze mainly homozygous populations, we attempted to genotype a highly heterozygous F2 population. We show how species- and population-specific improvements of established protocols can drastically increase sample throughput and genotype quality. Using as few as 50,000 reads for some individuals (134,000 reads on average), we were able to generate up to 8154 informative SNP markers in 1081 F2 individuals. Additionally, the effects of enzyme choice, read coverage, and data postprocessing are evaluated. Using GBS-derived markers, we were able to assemble a genetic map of 1536 cM. To demonstrate the usefulness of our GBS pipeline, we determined quantitative trait loci (QTL) for the number of tillers. We were able to map four QTL to chromosomes 1, 3, 4, and 8, and partially confirm their effects using introgression lines. We provide an example of how to successfully use GBS with heterozygous F2 populations. By using the comparatively low-cost MiSeq platform, we show that the GBS method is flexible and cost-effective, even for smaller laboratories.

Advances in sequencing technology have drastically improved our ability to determine and simultaneously genotype genetic markers (Davey *et al.* 2011). The enormous number of short (50–200 bp) reads produced by sequencing platforms has drastically reduced the costs and time associated with DNA sequencing. Those advances may be utilized in whole-genome resequencing approaches to generate a collection of reads from untargeted sites in the genome (Takagi *et al.* 2013; Duitama *et al.* 2015). Other approaches aim at reducing the complexity of the genome by sequencing only a targeted fraction of the genome. Such genotyping-by-sequencing (GBS) approaches were successful in generating tens of thousands of markers, even in plant species with large and repetitive genomes, like maize, wheat, or barley (Poland *et al.* 2012; Romay *et al.* 2013), or in more heterozygous animal species like cattle or pig (De Donato *et al.* 2013; Gualdrón Duarte *et al.* 2013).

It was shown that GBS can be used as a fast and cost-effective tool in population genetics, QTL (quantitative trait locus) discovery, high-resolution mapping, and genomic selection (Spindel *et al.* 2013; Rabbi *et al.* 2014; Huang *et al.* 2014; Burrell *et al.* 2015; Begum *et al.* 2015; Elmer *et al.* 2015; Lin *et al.* 2015). Since GBS data typically generate relatively dense marker data, a popular analysis choice is a genome-wide association study (GWAS) (He *et al.* 2014; Sonah *et al.* 2015; Begum *et al.* 2015). This kind of study employs a panel of cultivars or varieties. In addition, there are some examples of QTL analyses using biparental populations combined with GBS (Spindel *et al.* 2013; Honsdorf *et al.* 2014). In those studies, recombinant inbred lines that had already undergone several rounds of selfing were used to detect QTL. There are also examples of the use of GBS to genotype less fixed populations, like F2s (Rowan *et al.* 2015; Pootakham *et al.* 2015). In many cases, desirable traits are found only in wild relatives or are spread across diverse elite cultivars. The application of GBS to genotype F2s or breeding materials will greatly facilitate gene discovery and marker-assisted selection in breeding projects.

While GBS certainly has huge benefits for scientists and the breeding community, there are some inherent drawbacks to which no universal solution has yet been found (Poland and Rife 2012; He *et al.* 2014). The data produced by GBS, and similar strategies, has many missing datapoints compared to datasets from classical, “manually” produced genetic marker data or chip-based systems. Furthermore, there is a considerable error-rate associated with GBS-derived genotypes. Both of these issues can be dealt with at the cost of intensive postprocessing, data correction, and imputation, which is time consuming and requires specific bioinformatics attention. Also, for each GBS project, the researcher has to balance the cost of the sequencing platform with the goal of generating high enough read coverage, and, in turn, marker resolution for the intended analysis. Most GBS strategies aim to sequence only a defined fraction of the whole genome to reduce the number of reads necessary for adequate per-marker read coverage. A common approach is the use of one or two restriction enzymes (RE) to produce fragments with defined endpoints, instead of random shearing of input DNA. A recent protocol (Elshire *et al.* 2011) uses a combination of a RE with a 6 bp recognition sequence to target specific sites in the genome, and a RE with a more common 4 bp recognition sequence to generate fragments of suitable length. It was also shown that the choice of RE can influence sequencing results (Heffelfinger *et al.* 2014; Schröder *et al.* 2016). Another common strategy to reduce sequencing costs is the use of multiplexed libraries. By ligating a sample-specific, unique adapter sequence (also called a barcode) to the DNA fragments before pooling and library preparation, DNA from multiple individuals may be processed in a single library. Currently, between 96-fold and 384-fold multiplexed libraries seem to be most common, with between 500,000 and a few million reads dedicated to each individual sample.

In most cases, GBS aims to detect and simultaneously genotype a large number of single nucleotide polymorphism (SNP) markers. In this study, we used GBS on a rice F2 population derived from a cross of an elite cultivar from East Asia (*Oryza sativa* ssp. *Japonica* cv. Nipponbare, NB) and a West African wild rice (*O. longistaminata*, OL). Several complex traits are found in OL but are absent in NB. For example, OL is capable of perennial growth, while NB is an annual plant. Furthermore, OL is capable of clonal propagation through the use of rhizomes. To identify the genetic basis of those traits, we wanted to perform linkage analysis in an F2 population. Since there are only few markers available for this cross in public datasets, and traditional marker development and genotyping can be laborious, we established a GBS pipeline.

Performing GBS on an F2 population incurs some specific difficulties, since 50% of all SNP sites are expected to be in a heterozygous state. This demands higher read coverage to accurately call genotypes, since correctly calling a heterozygous allele requires the presence of reads from both allele states (Hyma *et al.* 2015; Johnson *et al.* 2015). Some existing GBS pipelines and imputation algorithms deal with that problem by omitting heterozygous calls. In our case that solution was not acceptable, since this would potentially eliminate 50% of all genotype information. Another problem associated with using a wild variant in a cross is that there is considerable heterozygosity in the wild parent’s genome. This can lead to the inability to correctly infer parental haplotypes. In addition, it might be possible that the wild parent (OL) has genome rearrangements, or gene copy number variations, as compared to the cultivated parent (NB). Those rearrangements might cause erroneous genotypes in specific regions and linkage of markers, which, in reality, are located on different chromosomes.

By a combination of the comparatively low-cost Illumina MiSeq platform (Loman *et al.* 2012) and high multiplexing, we created a flexible, medium throughput (a few hundred to 1000 individuals) genotyping pipeline. This approach is more flexible than hybridization-based assays (*e.g.*, Illumina’s Infinium Chips), can be adapted easily to new populations, and increased in scale to larger sequencing platforms. The pipeline was designed to specifically address rice F2 populations, but it should be useful for any F2 population. We investigated the effects of two different REs and different levels of multiplexing on the number of detected SNP markers. Also, we provide an example of how relatively low-coverage data (*ca*. 150,000 reads per sample) can be sufficient to generate high density genetic maps. Our pipeline uses simple error correction and imputation methods that take advantage of the long, uniparental haplotype blocks found in F2 populations. To show that our GBS pipeline is producing useful genotypes, we mapped QTL for tiller number, and partially confirmed these QTL using introgression lines derived from the same parents as the F2 population.

## Materials and Methods

### Plant cultivation and population development

The population used in this study was produced and cultivated in the International Rice Research Institute (IRRI), Los Baños, Philippines. An African wild rice, *O. longistaminata* Acc. IRGC110404 (OL) as male was crossed with the cultivar *O. sativa japonica* cv. Nipponbare (NB) as female to produce F1 plants, and subsequently F2 populations by self-pollination. Since NB and OL are rather distant relatives within the *Oryza* genus, there is some degree of incompatibility between both parents. Specifically, the cross between NB and OL led to a failure of endosperm development, resulting in embryonic death. Therefore embryo-rescue had to be performed to avoid embryonic death of F1 seeds. In total 301 and 813 F2 plants were grown in a paddy field enclosed by mesh (the screen house) to prevent insect damage at IRRI in the spring (Feb–May) and fall (Sep–Dec) seasons of 2014, respectively. The total number of tillers (primary and branched shoots of grass plants) was determined after digging up those F2 plants from the paddy field. Leaf blades of the F2s, and three replicate individual plants of each, NB and OL, were sampled for DNA extraction.

Previously, we developed a set of introgression lines (ILs) that harbor between one and three substituted genomic segments derived from OL in the NB genomic background (Ramos *et al.* 2016). The ILs consist of BC_4_F_7_ and BC_5_F_6_ plants derived from a cross between OL as female, and NB as male, and successive backcrosses by NB followed by self-fertilization. Four ILs were selected based on the QTL regions found in this study. The ILs and the recurrent parent NB were germinated in a greenhouse, and cultivated for 30 d. The seedlings were then transplanted to paddy fields at the research station of Nagoya University, Togo, Aichi Prefecture, Japan. Ten plants per line were planted in each row. The number of tillers was counted at the flowering stage in the ILs and NB, excluding damaged plants and plants next to the border of the plot to avoid position effects.

### Library preparation and sequencing

Genomic DNA from plant material was extracted using the cetyltrimethylammonium bromide (CTAB) method (Doyle and Doyle 1987). DNA integrity was analyzed by electrophoresis using a 1% agarose gel. DNA concentration of each sample was measured using a QuantusTM Fluorometer with a QuantiFluorTM dsDNA system (Promega, Madison, WI), and adjusted to 10 ng/μl. Libraries were prepared using a combination of two restriction enzymes according to (Poland *et al.* 2012) with the following modifications: genomic DNA samples (100 ng each) were digested in 20 μl of CutSmart Buffer by eight units of *Pst*I or *Kpn*I, each with eight units of *Msp*I [all New England Biolabs (Ipswich, MA); for *Pst*I and *Kpn*I the High-Fidelity version was used]. The digestion was performed at 37° for 1 hr, followed by an inactivation step at 65° for 20 min. Ligation was conducted in CutSmart Buffer without any modifications to the original protocol. A set of 192 unique barcodes were selected from the list of 384 barcodes designed for *Pst*I listed in Poland *et al.* (2012). These barcodes were utilized for both adapters with *Pst*I overhang and *Kpn*I overhang. Then 32-multiplexed libraries for samples digested by *Pst*I and *Msp*I, or 96-multiplexed libraries for *Kpn*I and *Msp*I, were prepared by pooling samples and subsequent PCR-amplification. DNA qualities and fragment sizes in the prepared libraries were evaluated using a Microchip Electrophoresis System for DNA/RNA analysis (MCE-202 MultiNA, SHIMADZU, Kyoto, Japan). In total, 10 32-multiplexed libraries and nine 96-multiplexed libraries were prepared. The libraries were sequenced using a MiSeq instrument with the MiSeq reagents kit v3 for 150 cycles (Illumina Inc., San Diego, CA).

### Detection of SNPs from raw sequencing data

To detect informative SNPs from raw sequencing data, the TASSEL 4 (Trait Analysis by Association, Evolution and Linkage 4) GBS pipeline (Glaubitz *et al.* 2014) was used (Supplemental Material, File S1). This included creation of a collection of unique, 64 bp long sequences (tags) from the raw sequencing data, alignment of tags to the IRGSP V1.0 *O. sativa* Nipponbare reference genome (Kawahara *et al.* 2013) using BWA (Burrows-Wheeler Aligner) (Li and Durbin 2009) with the –aln and –samse options, SNP calling, and filtering of SNPs based on minor allele frequency. To identify samples with poor read coverage, the TASSEL 4 log files for each library were inspected for individuals with very low read coverage (<1000 reads in our case). These individuals were removed from the analyses, or resequenced in another library if enough plant material was available. We noted that there is a positive correlation between the number of reads and the integrity of the extracted DNA. Initially, SNPs were called without specifying a filter using the DiscoverySNPCallerPlugin from TASSEL 4. Then, all SNPs with a minor allele frequency of <0.25 were removed, as those likely represented sequencing errors or rare alleles.

In the next step, the SNPs were filtered based on parental alleles to leave only SNPs which have fixed, but alternate alleles at any given locus. To achieve this, we selected only those SNPs which were: (1) not variable within each set of triplicate parental samples, (2) not heterozygous in either parent, and (3) different between both parents. Filtering was performed using the hapmap-formatted files and awk. The resulting collection of SNPs was then thinned out using vcf-tools (Danecek *et al.* 2011) to a minimum distance of 64 bp between two SNP sites. This eliminated redundant SNPs originating from the same tag, which, in most cases, had identical parental genotypes within each tag. This collection of SNPs was then used to explore the effects of different levels of missing data and imputation.

Preliminary analyses indicated that a large source of error would be undercalled heterozygous alleles (true heterozygous alleles wrongly called as homozygous alleles due to the absence of reads from one of the two states of a heterozygous allele). To counter this, we used vcf-tools to only allow genotypes that were supported by at least seven reads per site and sample. This limits the probability of undercalling a heterozygous site to a theoretical maximum of 1.6% (Swarts *et al.* 2014). Following this step, we directly compared the number of reads thought to originate from each parent at each heterozygous site. We calculated the relative allelic depth as the ratio of NB reads/OL reads, and found that >90% of all heterozygous genotypes had a <2-fold difference between the number of reads from each parent. This result indicated that there is no particular bias for reads from one of the parents (*e.g.*, different PCR efficiencies) that might have led to miscalled heterozygous genotypes. In the next step, a filter for different levels of missing data was implemented. Specifically, we generated (preimputation, pre-error-correction) datasets, in which up to 5, 50 or 75% of all genotypes for any given site were missing (File S2).

To rule out errors in data processing, we also directly analyzed alignments of reads to the Nipponbare reference genome independently from our GBS pipeline. For this, reads were demultiplexed using fastq-multx (https://github.com/brwnj/fastq-multx), and aligned using bwa as described above. We then inspected read alignments from selected, error-prone markers using the R package Gviz (Hahne and Ivanek 2016). When we analyzed the 10 F2 individuals that showed the highest number of single heterozygous genotypes flanked on both sides by Nipponbare alleles (AHA-type error, for more details see “*Results*”), we found that raw read alignments supported the called parental genotype in all analyzed cases.

### Imputation and error correction

As shown here, and in Spindel *et al.* (2013), GBS data inherently contains errors, and has to be imputed to be useful for linkage analysis. For our work, we took advantage of the fact that missing data, and wrongly called alleles, are distributed randomly across sites and samples. Furthermore, the F2 population in this study is characterized by long-range, uniform parental haplotypes that are long compared to the putative errors. We thus developed a simple imputation and error correction algorithm that is based on regular expressions and executed in R (R Development Core Team 2008).

In the first step, the data are transformed from the nucleotide-based hapmap format to an ABH-based format, where A represents NB, B represents OL, and H represents heterozygous alleles. After conversion, we first imputed missing data. Stretches of missing genotypes were filled with the appropriate allele if both flanking, not missing alleles were of the same state (*e.g.*, the sequence A**NNN**A, with A being a parental allele and **N** being missing data, would be imputed to A**AAA**A). Imputation of missing genotypes was not restricted by the length of each imputed stretch. *Post hoc* analyses showed that the maximum length of a stretch of consecutive imputed genotypes was seven, but >99% of all imputations were restricted to stretches of one or two genotypes (Table S1). The median imputed distance between two known genotypes varied between 571 kb (fall 2014 dataset, up to 75% missing data) and 1661 kb (fall 2014 dataset, up to 5% missing data). This imputation resulted in an almost complete elimination of missing alleles. Next, we tried to address the undercalling of heterozygous sites. Empirically, we set a minimum haplotype length of four sites. In any given F2 individual, if a series of homozygous or missing sites of length ≤4 was flanked on both sites by a heterozygous allele, this stretch was replaced with heterozygous sites. The other main error type seemed to be single erroneous alleles interspersed in longer homozygous haplotypes. We assumed those errors are cause by structural differences in the genome of OL compared to the NB reference genome. To counter this, we used a similar strategy as the one used to correct undercalled heterozygous alleles, but used a minimum haplotype length of one. This procedure reduced the number of missing genotypes as a percentage of all genotypes from 2.07 to 0.18%, while it increased the number of heterozygous alleles from 46.27 to 54.57% (data from the fall 2014 population, with up to 75% missing data per site, full dataset in Table S2). In the final step, data from both analyzed populations was combined based on the assumed physical position of SNP markers. Since two different enzymes were used for the spring 2014 and the fall 2014 population, no SNP marker was found in both datasets, as different enzymes generate different sets of reads. Thus, we imputed missing data again, using the rules devised above to fill in sites. This imputation step was characterized by a median length of consecutive imputed genotypes of six, and a median length of imputed distance between genotypes of 0.86 Mbp when the missing *Pst*I-derived genotypes (more common) were imputed in the *Kpn*I-derived genotypes (less common) in the datasets with up to 5% of missing data (Table S1).

Our imputation method relies on identical alleles in two flanking markers to make predictions about the genotypes in between. This approach does not take into account rare double crossover events between those markers. We estimated the possibility for a double crossover event to occur within 1 Mb to be ∼0.15%. This is based on a genetic map with a total length of 1536 cM and a physical length of 400 Mb, which can be expressed as 3.84 cM/Mb. The probability for a double crossover event within 1 Mb was thus calculated as 0.0384^2^ = 0.0014756. Given that median imputed distances in the two imputation steps were mostly below 1 Mb, we concluded that our imputation will miss a double crossover event in approximately <1 out of 1500 imputation events.

All TASSEL scripts, and the scripts used for post-TASSEL data processing, can be found in File S1. The imputation and error correction logic described here (in addition to functions for graphical analyses of genotypes) is also available in the “ABHgenotypeR” package for R, which is available at https://github.com/StefanReuscher/ABHgenotypeR, or via CRAN (Comprehensive R archive network).

### Confirmation of genotypes by Sanger sequencing

Ten error-prone SNPs identified by the GBS pipeline were selected for Sanger sequencing (Table S3). PCR primer sets to amplify a 150–300 bp region around the selected SNPs were designed based on the IRGSP V1.0 *Oryza sativa* Nipponbare reference genome sequence (Table S4). Genomic DNA extracted from NB, OL and 10 F2 samples with a high error rate were subjected to PCR for the region of the selected SNP. The PCR products were purified by gel purification using the Wizard SV Gel and PCR Clean-Up System (Promega, Madison, WI). After confirming the presence of a single PCR product using 1.5% agarose gels, Sanger sequencing was performed with either of the PCR primers as a sequencing primer using BigDye Terminator v3.1 Cycle Sequencing Kit (Applied Biosystems) and a 3130xl Genetic Analyzer (Applied Biosystems, Foster City, CA). The sequence data, including the raw chromatograms, was analyzed using the sequence assembly software ATGC (Genetyx Corporation, Tokyo, Japan) to identify nucleotides at the SNP positions detected in GBS.

### Data analysis

General data analysis was performed using the TASSEL graphical user interface, and R. QTL analyses and simulations were performed using the R package “qtl” (v1.37.11) (Broman *et al.* 2003). For QTL simulations, phenotypic values and genotypes of simulated F2 populations were generated using the function “sim.cross” implemented in the “qtl” package and described in detail in Broman and Sen (2009). “sim.cross” requires a genetic map of markers, the number of individuals, and a model of QTL to generate a simulated population. For simulating the genetic map of markers, we used the “sim.map” function, which requires chromosome lengths and marker numbers. The lengths of the chromosomes were set to 140, 115, 130, 110, 100, 105, 110, 100, 75, 80, 100, and 105 cM for chromosomes 1–12, respectively, based on a genetic map of microsatellite markers developed in our previous QTL study for F2 populations derived from a cross between NB and OL. Simulations with 50, 100, 200, or 400 equally spaced markers were performed. For simulating phenotypic values that were affected by a number of simulated QTL, we assumed the existence of eight QTL (on 8 out of 12 chromosomes), each of which had an additive effect of 0.5. The residual phenotypic variation was assumed to be normally distributed with a variance of one. Under these assumptions, each of the simulated QTL had 4.17% contribution to the phenotypic variance. With the simulated genetic map and the QTL model, data sets of F2 populations for 200, 400, 600, 800, and 1000 individuals were generated using “sim.cross.” We performed simple interval mapping in the simulated F2 populations using the function “scanone,” with the multiple imputation method (Sen and Churchill 2001). In the multiple imputation method, genotypes between markers were imputed with 1 cM intervals based on genotypes of flanking markers, and multiple imputed genotype data were generated for each individual. Then, a linear regression model was fitted for each marker using the imputed genotype data, and the phenotype data with the assumption of normal distribution of phenotypic values. The threshold for significant LOD scores was calculated from 1000 permutation tests. According to past studies, confidence intervals of detected QTL were usually >10 cM (Kearsey and Farquhar 1998; Darvasi 1998), so we used that size as a threshold. If a significant QTL (*P* ≤ 0.05) was detected around the simulated, true QTL position (±10 cM), we counted it as correctly detected. For each condition, 100 simulations were performed, and the probability to correctly detect all QTL was calculated.

Genetic maps using real data were constructed using the “est.map” function with default parameters. QTL analyses for the number of tillers in 1081 F2 plants was performed using a linear regression model with the multiple imputation method by “scanone.” The threshold for significant LOD scores was calculated from 1000 permutation tests. The 95% confidence intervals of significant QTL were estimated using the function “bayesint,” which takes 10^LOD score^ values for an obtained LOD profile and rescales it to have an area of one, followed by calculating the connected interval having 95% coverage of the area. The function “fitqtl” was used for calculating percentages of variance of the significant QTL by calculating the coefficient of determination for each single-QTL model obtained using “scanone.” Additive and dominant effects of the significant QTL were calculated from mean phenotypic values for each genotype at the QTL positions obtained by using the function “effectplot.”

Genome-wide analysis of restriction sites was performed using the “restric” tool from the emboss software suite (Rice *et al.* 2000). Random sampling of reads from fastq files was performed using fastq-tools (http://homes.cs.washington.edu/∼dcjones/fastq-tools/).

### Data availability

File S1 contains all code necessary to replicate the GBS-pipeline. The data imputation and error-correction logic is also available in the R package “ABHgenotypeR.” File S2 contains all genotypes from this study, including marker order and position. All demultiplexed reads used for SNP calling were submitted to the DNA Data Bank of JAPAN (http://www.ddbj.nig.ac.jp/), and are available under bioproject PRJDB5346. 

## Results

### Application of GBS to a rice F2 population

**Figure 1:**
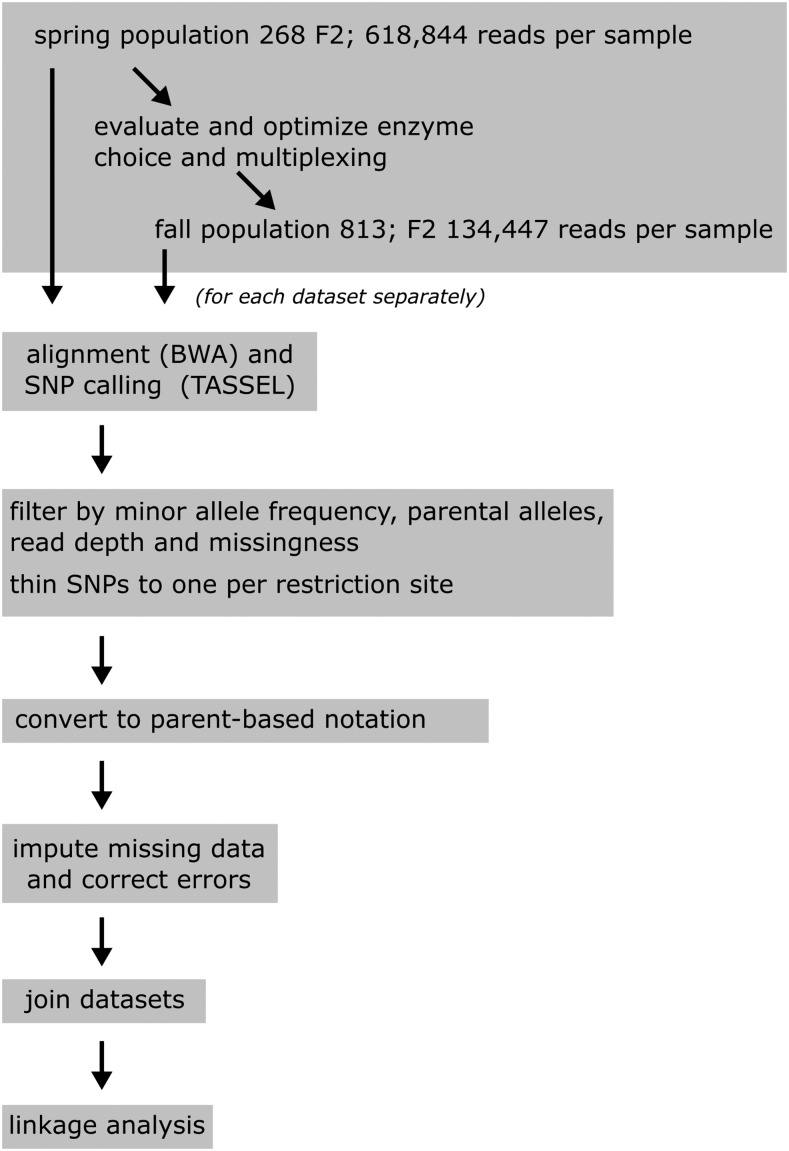
Flowchart of the GBS data processing. A schematic overview of the different steps of the GBS pipeline.

A population of 268 F2 plants from a cross of NB and OL, including triplicate parental samples, from the spring 2014 season was sequenced first. From this population, libraries of 32 samples each were prepared, and processed with the GBS pipeline (Figure 1). This approach resulted in 618,844 average reads per individual, which yielded (with up to 5% missing data) 2144 SNPs (Table 1). Analyses using simulated data to determine QTL detection probabilities showed that this number of markers is more than sufficient to detect even weak QTL (see Figure S1). In fact, a few hundred markers gave sufficient detection power, while, at the same time, the number of F2 individuals appears to be the limiting factor. We therefore optimized our GBS pipeline to process more F2 individuals, at the expense of generating a lower number of SNP markers, by multiplexing more samples per library.

**Table 1:** Basic parameters of both GBS experiments described in this work

	Spring 2014	*Fall 2014*
Enzymes	*Pst*I-*Msp*I	*Kpn*I-*Msp*I
Number of F2 individuals	268	813
Multiplexing	32	96
Reads per sample (mean ± SD)*^a^*	618,843.7 ± 178,135.8	134,447.3 ± 50,788.87
No. of sites (<5% missing data)	2144	301
No. of sites (<50% missing data)	5812	837
No. of sites (<75% missing data)	7058	1096

a Numbers are based on good, barcoded, aligned reads.

For a larger population of 813 F2 plants, and triplicate parental samples from the fall 2014 season, the following changes were implemented: (1) instead of using *Pst*I as the rare-cutting enzyme, we used *Kpn*I. There are 107,953 *Pst*I cut sites reported in the NB reference genome, while there are only 45,065 *Kpn*I cut sites according to *in silico* digests. Thus, if all parameters were kept constant, in libraries prepared with *Kpn*I, the resulting reads will be distributed among fewer sites, but reach a higher per-site coverage. (2) Taking advantage of the higher per-site coverage using *Kpn*I, we increased the number of samples per library. Prior to library preparation, we examined the effects of decreased read coverage per F2 individual by randomly sampling a fraction of reads from each input fastq file. In these simulated multiplexing analyses, it became clear that the undercalling of heterozygous sites (50% in an F2 population) would become a large source of errors if multiplexing is increased (see Figure S2). Based on those results, 96-fold multiplexing was deemed feasible, and was implemented with the fall 2014 population. This resulted in an average of 134,447 reads per F2, which yielded (with up to 5% missing data) 301 SNPs (Table 1).

As expected, higher multiplexing, and a change to *Kpn*I, led to a lower number of detected SNP sites. When processed through our GBS pipeline, however, both datasets led to similar genotype patterns, the main difference being the number of sites that were reliably detected. As the final step of the GBS pipeline, both datasets were merged. To describe and evaluate the results of the GBS pipeline, we subsequently used data from the fall 2014 dataset. For results regarding the genetics of the NB × OL F2 population and linkage analysis, we used the combined datasets to maximize detection power and resolution.

### Analysis of general SNP properties

**Figure 2:**
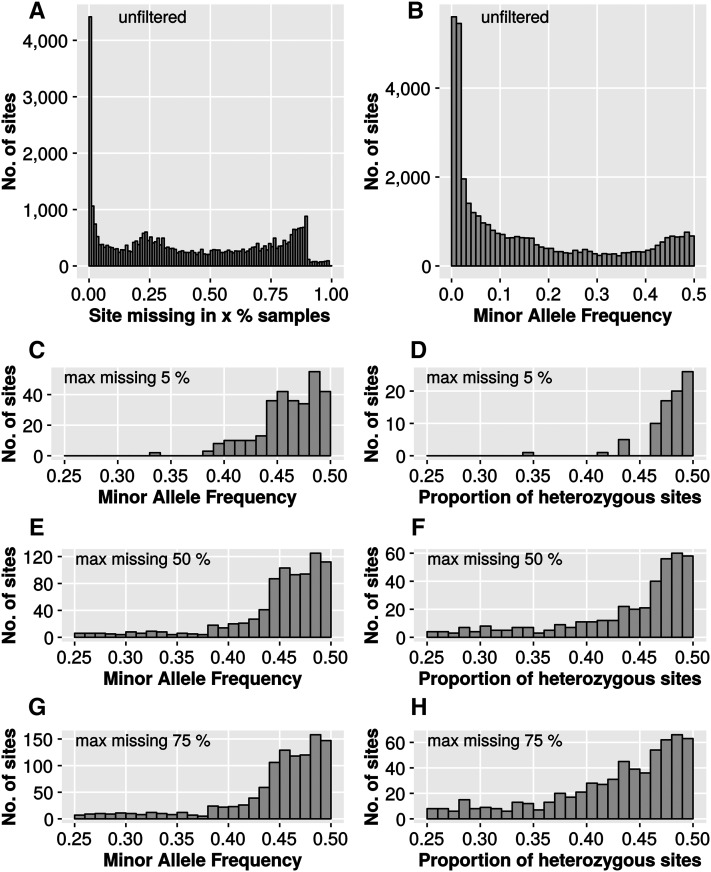
Basic SNP characteristics using different filter settings for missing data. Shown are histograms representing the number of SNP sites that exhibit a certain sample coverage (A), minor allele frequency (B, C, E, and G), or proportion of heterozygous sites (D, F, and H). Data are from 813 F2 plants from the fall 2014 population, and were generated using the TASSEL 4 site report function. For (A) and (B), unfiltered data directly after SNP calling was used. For (C)–(H), SNP sites were filtered by the indicated proportion of missing data per sample, but no further data imputation or error correction was performed.

The unfiltered GBS dataset contained a high proportion of missing data (Figure 2A) and only ∼4500 out of 37,938 sites were detected in all samples. Also, a substantial number of SNPs was observed, with very low minor allele frequencies (MAF) (Figure 2B). We used a threshold of MAF >0.25, and different proportions of missing data (<5, <50, <75%), and analyzed the MAF and the proportion of heterozygous sites. When using a very stringent filter of <5% missing data, both the MAF and the proportion of heterozygous sites reached a lower limit at ∼0.35 (Figure 2, C and D). At a higher proportion of missing data, some sites could be observed that had a MAF and proportion of heterozygosity as low as the set threshold of 0.25 (Figure 2, E–H). The bigger spread in allele frequencies and heterozygosity observed for datasets with a higher percentage of missing data might be explained by the inclusion of sites with low read coverage in those datasets. SNP sites that are supported by a small number of reads are more prone to errors. For example, reads representing either NB or OL alleles could have different amplification efficiencies during library preparation. For SNPs with high read coverage this might have no effects, but, for SNPs with low read coverage, this might skew our ability to detect a specific allele. This observation highlights the importance of both adequate read coverage and post SNP-calling error correction.

To evaluate the fidelity of GBS genotypes, we independently genotyped 93 F2 plants using simple-sequence repeat (SSR) markers, and compared both sets of genotypes. It was found that the majority of parental genotypes (>90%) was identical when the results of both genotyping systems were compared (see Figure S3). The 10% disagreeing markers are explained by single SSR markers, in which up to 1/3 of all genotypes disagree, probably by the SSR marker and the closest GBS marker being on different sides of a recombination event.

**Figure 3:**
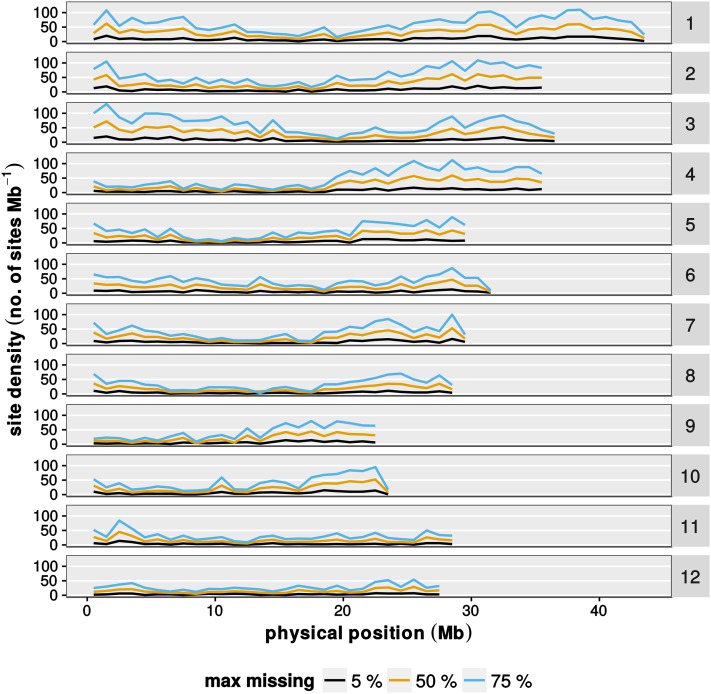
Marker densities along the chromosomes. Shown is the marker density along the 12 rice chromosomes. The number of markers was determined for bins of 1 Mb. Different colored lines represent datasets with the indicated proportion of missing data. Data are from the fall 2014 population (*n* = 813 individuals).

Next, we evaluated the distribution of SNP sites along the chromosome (Figure 3). SNP sites were notably sparser in the centromeric regions, probably as a result of a high amount of repetitive sequence elements, which prevent reads to be mapped to a unique position. Also, the distribution of sites along the chromosome arms was not even. In general, the SNP density at any given chromosome position increased with the amount of missing data allowed. However, there were some chromosomal regions with low SNP density in which the number of SNPs was hardly affected by the amount of missing data. This was not caused by uneven distribution of *Kpn*I recognition sites (data not shown). For example, a SNP density below the average was observed on the long arms of chromosome 4 and chromosome 9. The occurrence of such SNP deserts was observed before (Wang *et al.* 2009; Krishnan *et al.* 2014), but it is unclear if and how those regions are associated with domestication. Lower than average SNP density might also be caused by methylation sensitivity of the *Msp*I enzyme used during library preparation. *Msp*I cannot cut its CCGG recognition sequence if the external C is methylated. This might lead to an under-representation of SNPs from heavily methylated regions, such as the centromere, or other repeat-rich regions.

**Figure 4:**
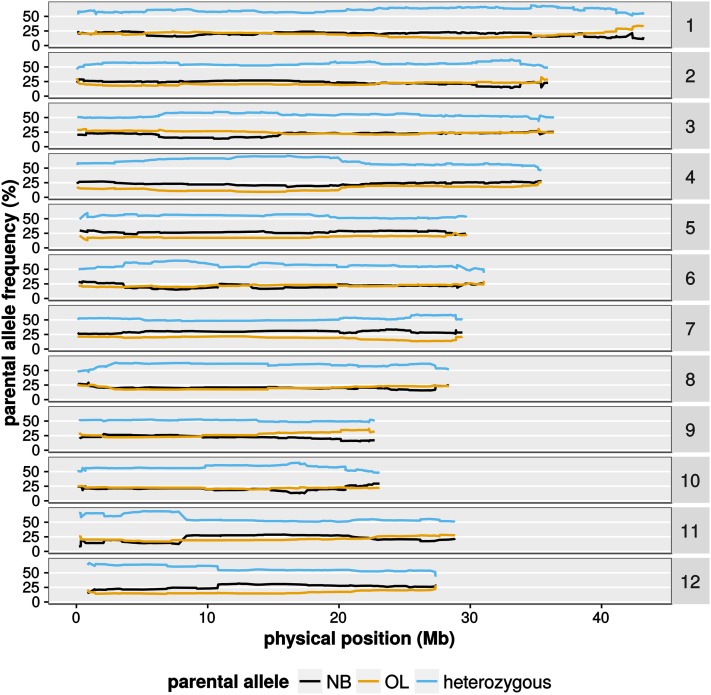
Parental allele frequencies along the chromosomes. Shown are the frequencies of parental alleles observed along the 12 rice chromosomes. Data are from the joined datasets from spring and fall 2014. Only markers present in 95% of all samples in the respective dataset are shown.

In an ideal F2 population, one would expect that the parental alleles segregate according to a 1:2:1 ratio (parent A: heterozygous: parent B). However, a plot of allele states along the chromosomes revealed regions with distorted genotype ratios (Figure 4). As a general trend, the OL alleles seemed to be transmitted at slightly lower levels. As an extreme example, the long arm of chromosome 4 has a drastically reduced frequency of the OL allele, with OL genotype frequencies decreasing to <10%, as opposed to the expected 25%. In most chromosomal regions where one parental allele was found under-represented, the frequency of heterozygous genotypes in turn was increased to >50%. Very likely, those effects are due to chromosomal regions associated with reproductive incompatibility.

### Constructing a genetic map

**Figure 5:**
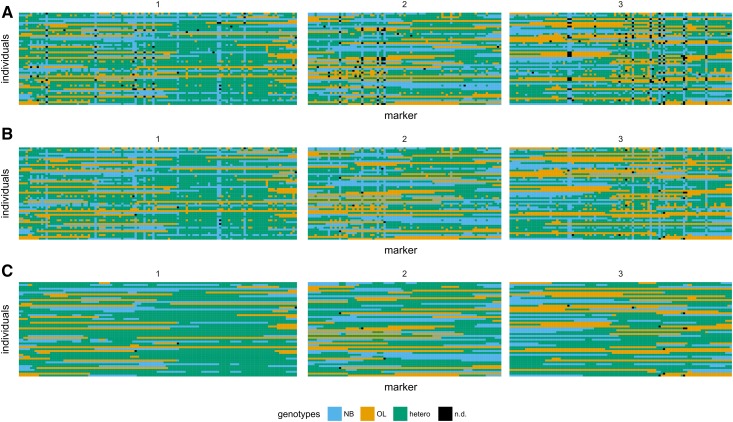
Graphical representations of GBS-derived genotypes at different stages of post-processing. Shown are graphical representations of genotypes after inferring parental alleles (A), after inferring parental alleles and imputation of missing data (B), and after inferring of parental alleles, imputation, and error correction (C). Genotypes of 50 representative F2 individuals are shown, with each F2 as a single horizontal track. The chromosome length is proportional to the number of markers, and only chromosomes 1 to 3 are shown. In total, 312 markers (fall 2014 population, up to 50% missing data) are displayed, with genotypes color-coded as blue (NB), orange (OL), green (heterozygous), and black (not determined).

To inspect GBS genotypes and haplotypes, we constructed graphical representations of genotypes (Figure 5, full dataset in File S2). This made it obvious that GBS data without imputation and error correction contains wrongly called genotypes (Figure 5A). Since F2 populations have relatively long haplotypes, the observed very short (1–2 markers) uniform genotype stretches found as islands in longer stretches are most likely errors. After imputation of missing data (Figure 5B), we used a simple error correction algorithm based on haplotype length to efficiently correct those errors (Figure 5C).

**Figure 6:**
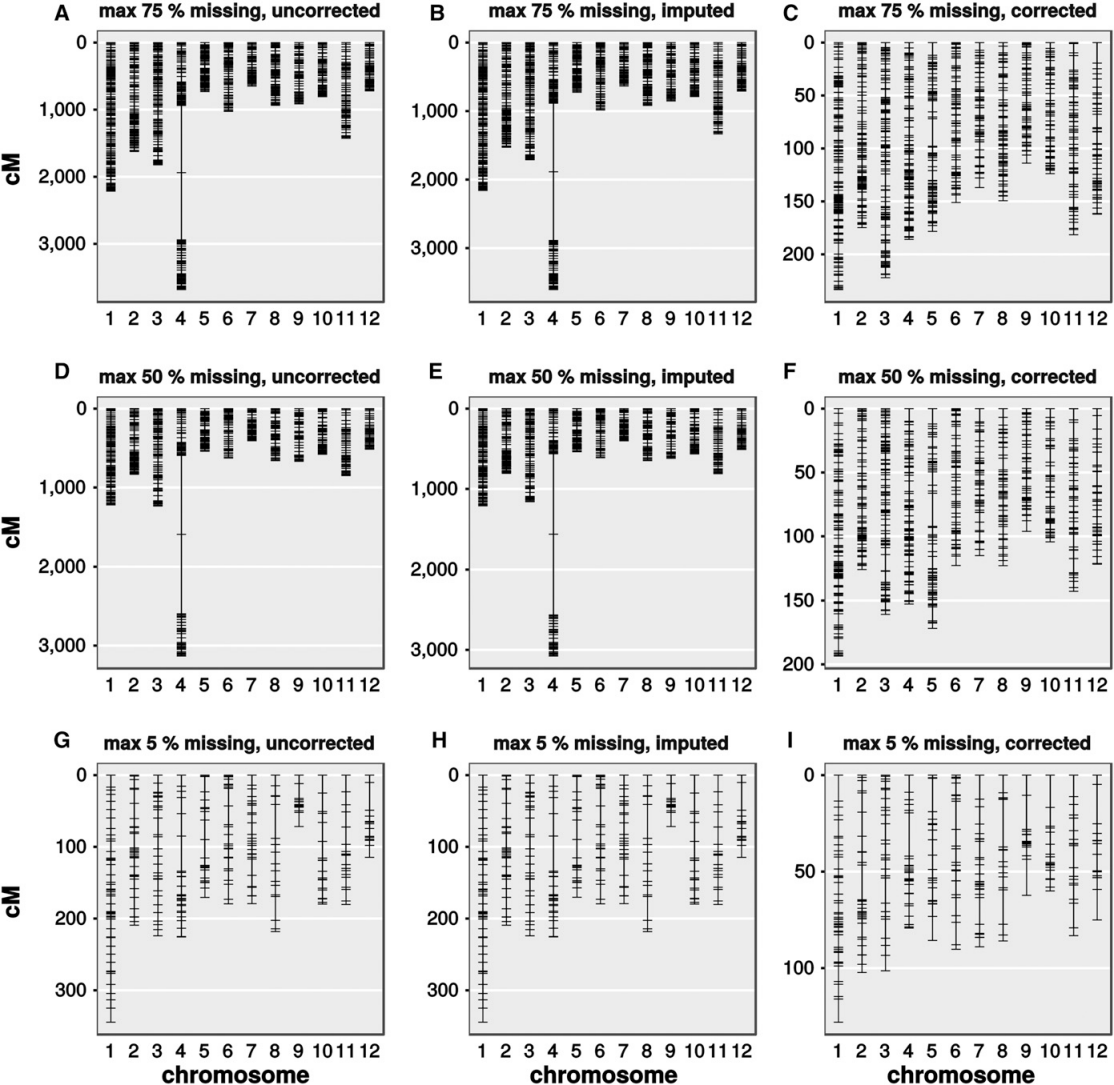
Genetic maps from datasets with different proportions of missing data and post-processing. Shown are linkage maps of GBS marker datasets. Panels show datasets with SNP-calling thresholds allowing up to 75% (A–C), 50% (D–F), and 5% (G–I) missing data, at different steps of the GBS pipeline. Uncorrected (A, D, and G) indicates data without further postprocessing. Imputed (B, E, and H) indicates data with missing data imputed, but no error correction performed. Corrected (C, F, and I) indicates data with both imputation and error-correction performed. Data are from 813 F2 plants from the fall 2014 dataset. Distances between markers are shown in cM.

When we used the fall 2014 dataset to construct a genetic map, it became again clear that raw GBS data cannot be used directly (Figure 6). When uncorrected data with up to 75 or 50% (Figure 6, A, B, D, and E) of missing data per site was used to generate a genetic map, chromosomes appeared expanded with chromosomes of up to 3500 cM. The map distention we observed was conspicuously similar to the distention shown in Spindel *et al.* (2013), and we applied a similar strategy to consolidate our genetic map. Both a rigorous restriction on missing data (up to 5% missing, Figure 6, G–I) or imputation and error correction (Figure 6, C and F) seemed to alleviate the problem. Restricting missing data led to a strong reduction of available SNP sites (compare 837 for 50% missing to 301 for 5% missing), but also shortened the genetic map. Using filtering, imputation, and error correction, we gradually improved the genetic map, even when up to 75% of genotypes were initially missing for each individual site. The final genetic map (Figure 6I) had a total size of 1536 cM, which is in agreement with other data. We still observed some distention, for example on chromosome 5 and chromosome 12. Although haplotypes and alleles appear to be correct in those regions, we can observe strong linkage of markers in those regions with markers from different chromosomes (data not shown).

### Investigating error-prone SNP markers

Although we were able to correct most assumed errors in the parental genotypes, and construct a reasonable genetic map, we further analyzed the types of errors that occurred. We first defined all haplotypes of length = 1 as errors; *e.g.*, a single A (representing NB) flanked by B genotypes (representing OL), as this is very unlikely to reflect the true allele state in an F2 population at the given marker density. Using the imputed, but not error-corrected, data we counted the occurrences of each possible error-type (HAH, HBH, AHA, BHB, ABA, and BAB) in the dataset from fall 2014 (5% max missing) for each of the 301 SNPs, and for each of the 813 F2 individuals, respectively (Table S3).

**Figure 7:**
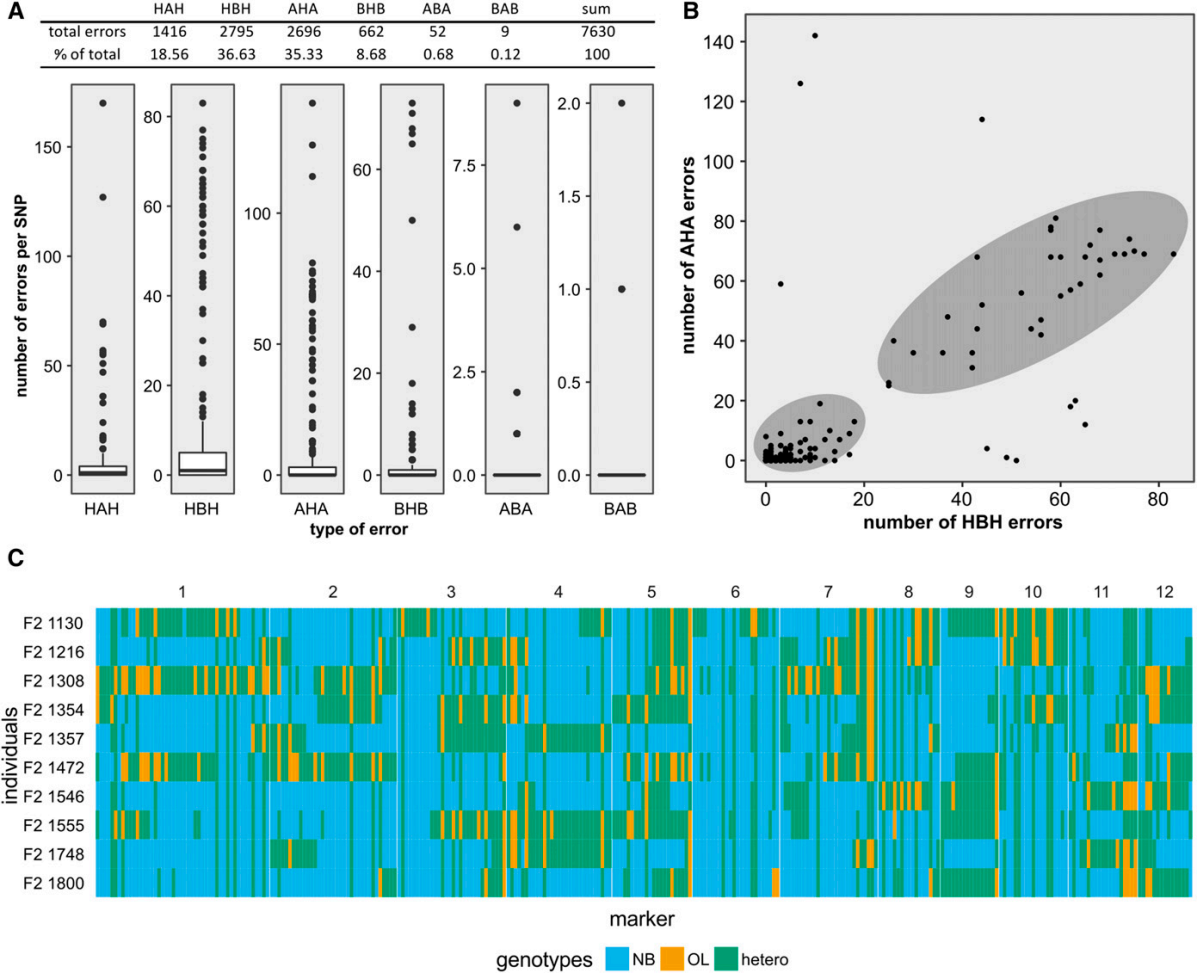
Analyzing types of putatively wrong allele calls. The distribution of all six possible error-types is shown as a boxplot (A). In (B), each point represents one SNP plotted according to the two most prevalent error-types (HBH and AHA). The scatterplot revealed two distinct clusters of error frequencies indicated by the two shaded regions. In (C), graphical genotypes are shown as described in Figure 5. Data from 10 F2 with the most AHA errors are shown. Uncorrected data from the fall 2014 dataset (maximum missing 5%) are shown in all panels.

We first analyzed the distribution of the occurrence of each possible error-type per SNP (Figure 7A). This distribution made clear that most SNPs have very few (75%-tile ≤5) errors (as defined above). However, for HAH, HBH, AHA, and BHB-type errors, a small number of SNPs accounted for most of the errors. The two most frequent types of errors were “undercalled” OL alleles (HBH, 36.63% of all errors), and “overcalled” heterozygous alleles within a NB context (AHA, 35.33% of all errors). When we performed a scatterplot analysis using the number of HBH and AHA errors (Figure 7B), the SNPs formed two distinct clusters. SNPs in the low-error cluster were correctly called most of the time. SNPs in the high-error cluster tended to accumulate both HBH and AHA errors at the same time. This observation was confirmed when we visualized uncorrected genotypes from 10 F2s in which the most AHA-type errors were detected (Figure 7C). In those F2s, some markers consistently showed HBH- and AHA-type errors. This hints at a hidden, common cause for those two error-types.

In the next step, we analyzed whether obvious genomic rearrangements might be the cause for error-prone SNPs. To this end, we isolated a 65 bp sequence from the NB reference genome centered on the location around the SNP. This sequence was then used as a query for a BLASTN search in the recently published OL genome (Zhang *et al.* 2015), retaining the best three hits. Out of 301 sequences covering the SNP positions, 206 had their best BLASTN hit on the same chromosome in both NB and OL, indicating that there is a syntenic relationship (Table S3). For 27 sequences, no BLASTN hit was found, which is probably due to the lower coverage of the OL genome compared to the NB genome. For 68 sequences, however, the best BLASTN hit in the OL genome was on a different chromosome when NB and OL were compared. In addition, for 28 out of the 206 syntenic markers, BLASTN reported at least one additional hit. When we combined the data from BLASTN searches with the occurrences of overcalled AHA alleles per marker, we found that the top three most error-prone markers appeared to be nonsyntenic in NB and OL. However, from the top 10 most error-prone markers, only four appeared to be nonsyntenic, while six appeared to be syntenic. This indicated that nonsyntenic markers might explain some of the observed errors, but they cannot explain all observed errors.

In addition to using SSR markers (Figure S3), we also used Sanger sequencing to determine the nucleotides at SNP positions. We sequenced the genomic region around 10 particular error-prone SNP sites in 10 F2 plants and both parents. This enabled us to directly compare GBS- and Sanger sequencing derived genotypes (File S3). For the 10 analyzed F2 plants, 7 out of ten SNPs showed identical parental alleles between GBS and Sanger sequencing derived genotypes, despite many of those genotypes forming haplotypes of length = 1, and thus being flagged as errors as described above. This result rules out that mere technical errors (wrong base calls, misaligned reads) are a major source of seemingly erroneous alleles. Instead, it is possible that structural differences between the parental genomes are a major source of error in this population. To still allow the calculation of a genetic map for later QTL analysis, we assumed haplotypes of length = 1 to be errors and corrected them as described before.

### QTL analysis

**Figure 8:**
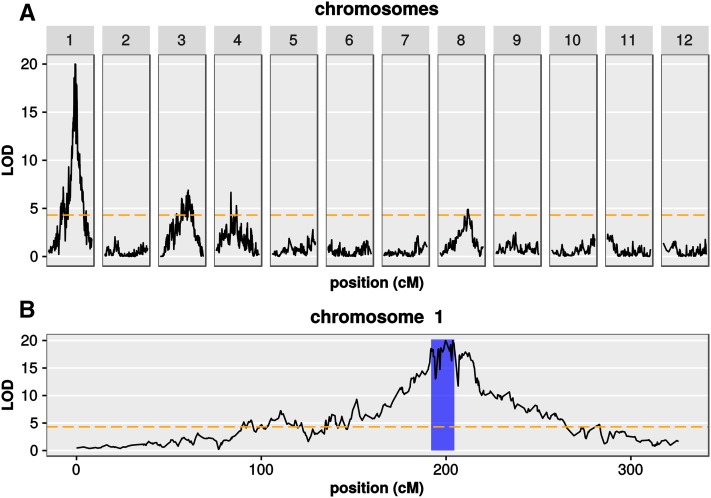
Detection of QTL for tiller number using GBS markers. Shown are the results of a linkage analysis to detect QTL that have an effect on tiller number using data from joined spring and fall datasets with up to 75% missing data per marker. LOD scores are shown as black lines for all 12 chromosomes (A), or for chromosome 1 only (B). A LOD threshold for significance (*P* ≤ 0.05) is shown as a dashed orange line. The blue area in (B) highlights the 95% confidence interval of qOLTN1 (QTL1 for tiller number *O. longistaminata*). Distances are shown in cM.

Being able to produce a correct genetic map using the combined dataset reassured us that our GBS data are sufficient for linkage analysis. For QTL analysis in 1081 F2 plants, we chose to use the number of tiller as the phenotype. We detected four significant QTL on chromosomes 1, 3, 4, and 8, which were named qOLTN1, qOLTN2, qOLTN3, and qOLTN4, respectively (Figure 8A). Among these four QTL, qOLTN1 on chromosome 1 showed the highest LOD score with 20.15 (Figure 8B), while the other QTL showed LOD scores <6.9. To analyze these QTL in more detail, we calculated 95% confidence intervals, percentages of variance, and effects for each QTL (Table 2). The confidence interval of qOLTN1 spanned a 3.6 Mb region from 27.1–30.7 Mb on chromosome 1. This QTL explained 8.23% of the variance in the number of tillers of the F2 population, and showed a negative additive effect of −9.17 and a positive dominant effect of 5.22. These results suggested that an OL allele at qOLTN1 acts recessive to decrease the number of tillers as compared to NB. Unlike the case of qOLTN1, the other QTL gave only little contributions on the differences in the number of tillers, and relatively smaller effects (Table 2). Interestingly, qOLTN4 exhibited a positive superdominant effect, in which the additive effect was −2.44, while the dominant effect was 4.51. This result means that heterozygotes at qOLTN4 produce more tillers than either NB homozygotes or OL homozygotes.

**Table 2:** Percentages of variance and effects of the significant QTL

QTL Name*^a^*	Chr	LOD Score	Left Bound*^b^*	Peak Position*^c^*	Right Bound*^d^*	% of Variance	Additive Effect*^e^*	Dominant Effect*^f^*
qOLTN1	1	20.15	27,085	29,323	30,648	8.23	−9.17	5.22
qOLTN2	3	6.67	16,459	23,610	27,706	2.80	−4.66	−0.65
qOLTN3	4	6.68	12,436	12,591	18,420	2.80	5.07	−1.10
qOLTN4	8	4.91	16,523	19,907	22,362	2.07	−2.44	4.51

a *O. longistaminata* tiller number.

b Chromosomal positions in kilobase of left bounds of the 95% confidence intervals. All chromosomal positions are based on the physical position of the closest marker in the NB reference genome.

c Chromosomal positions in kilobases, where the maximum LOD scores were detected for each QTL.

d Chromosomal positions in kilobases, of right bounds of the 95% confidence intervals.

e Positive values indicate increases of the number of tillers in OL homozygotes, while negative values indicate decreases in OL homozygotes compared to NB.

f Positive values indicate increased tiller number in heterozygotes compared with the averages of NB and OL homozygotes, while negative values indicates decreases in heterozygotes compared with the averages of NB and OL homozygotes.

To evaluate the results of our QTL simulation (see Figure S1) against this real data, we performed linkage analyses for random subsets of varying numbers of F2 plants. As predicted in our simulations, we found that up to 1000 F2 plants are necessary to reliably detect all significant QTL (see Figure S4). When we used all 1081 F2 plants for linkage analysis, but varied the amount of missing data allowed in the prefiltered datasets, we found very similar LOD score profiles (see Figure S5). We thus used the dataset with up to 75% missing data per site before postprocessing to maximize marker resolution.

To verify the QTL, we conducted a field experiment to measure the number of tillers in ILs having OL genomic segments at each of the QTL locations. Four ILs having OL chromosomal segments around QTL locations were selected from the pool of ILs, and named IL-qOLTN1, IL-qOLTN2, IL-qOLTN3, and IL-qOLTN4, for having OL chromosomal segments around qOLTN1, qOLTN2, qOLTN3, and qOLTN4, respectively. IL-qOLTN1 and IL-qOLTN2 showed a significant decrease in the number of tillers compared with NB (Table 3). The reductions of tillers in these two ILs are in agreement with the negative additive effects of qOLTN1 and qOLTN2 (Table 2). Furthermore, IL-qOLTN3 and IL-qOLTN4 produced more and fewer tillers than NB, respectively, although the differences were not significant. However, the results observed in IL-qOLTN3 and IL-qOLTN4 also corresponded to the positive and negative additive effects of qOLTN3 and qOLTN4, respectively. In summary, we could successfully detect four QTL using our GBS data for the number of tillers, and partially verify the effects of those QTL in ILs.

**Table 3:** Tiller number in the introgression lines

Genotype*^a^*	Chr*^b^*	Markers*^c^*	Position*^d^*	No. of Tillers*^e^*
NB	—	—	—	13.67 ± 1.70
IL-qOLTN1	1	RM1287-RM297	10.8–33.8	8.14 ± 1.64*^f^*
IL-qOLTN2	3	OL3L26-RM3436	5.4–28.2	11.13 ± 1.27*^f^*
IL-qOLTN3	4	End-RM3866	0–23.8	15.00 ± 3.12
IL-qOLTN4	8	RM1235-RM5485	12.1–24.2	11.67 ± 2.87

a NB indicates Nipponbare, IL-qOLTN1–4 indicates introgression lines that carry the respective QTL for tiller number.

b Chromosome which have an *O. longistaminata* chromosomal segment.

c Flanking simple sequence repeat markers of an introgressed *O. longistaminata* chromosomal segment. “End” indicates the end of short arm.

d Physical positions of the flanking SSR markers in megabases.

e The number of tillers measured in 7–9 plants for each line are shown in mean ± SD.

f Indicates a significant difference compared with NB at *P* ≤ 0.05 according to Student’s *t*-test.

## Discussion

Our aim for this study was to utilize GBS for rapid genotyping of rice F2 populations. As expected, GBS proved to be a robust and efficient method to genotype large populations (Elshire *et al.* 2011; Spindel *et al.* 2013; Lu *et al.* 2013; Liu *et al.* 2014). For successful application of GBS, it is necessary to generate adequate read coverage across the genome, and also for each individual that is sequenced. In our approach to genotype a rice F2 population, we further took into account the number of individuals and markers that are necessary to detect QTL. Since one of the main motivations to perform GBS is to save time and money compared to classical markers, one would like to use as few sequencing runs on any platform as necessary to achieve the desired sequencing depth. Our choice to change the enzyme from *Pst*I to *Kpn*I led to predictable changes in the resulting SNP collection. Other reports have also shown that enzyme choice is an important parameter to optimize GBS for any given species, highlighting the importance of using *in silico* digests of the genome of the target organism beforehand (Heffelfinger *et al.* 2014; Schröder *et al.* 2016). Marker density depends also partially on sequencing depth, which in turn depends on the number of individual per sequencing run. To be most efficient, it is thus advisable to take into account the desired marker density when laying out a genotyping project involving GBS. In our experience, performing a small-scale pilot experiment using the desired population and sequencing platform, combined with linkage analysis on simulated data, allowed us to use GBS more efficiently. The results of linkage analyses using both simulated (see Figure S1) and experimental (see Figure S4 and Figure S5) data suggested that our GBS approach resulted in a saturation of markers. The fact that our linkage analysis yields comparable results, even when up to 75% of missing data for each marker were acceptable in the raw data, shows that even simple imputation algorithms can reinforce the usefulness of GBS data tremendously. We speculate that, for certain applications, even fewer markers, and, in turn, fewer reads per individual would be sufficient, thus allowing even higher multiplexing and sample throughput. Of course, this might also depend on the genome size of the analyzed species, and the amount of repetitive elements in that genome.

### Optimizing GBS strategies

Although we were successful in using GBS, we noticed several shortcomings where no best practice seems yet to be established. There seems to be very little consensus about how GBS protocols should be adapted to different species, and to different populations. For variant calling, filtering, and exploration of our dataset we used the TASSEL4 (Glaubitz *et al.* 2014), which was develop to work efficiently with large maize populations. It became apparent that additional specific bioinformatics analyses were necessary to get the most information from our dataset. This shows that a given GBS protocol needs to be optimized for a specific species or population. Another issue is the high error rate of raw GBS data. While it is possible to eliminate most errors using post SNP-calling error correction, some errors will inevitably remain. Our results indicated that simple technical errors (*e.g.*, wrong base calls or misaligned reads) are not a major source of errors. It would thus be worth investigating the source of errors, as this might lead to new insights into the population in question. In our case, where a wild species is crossed to a cultivated one, it can be assumed that there will be large-scale differences between the two parental genomes contributing to the F2 individuals (Wang *et al.* 2014). Those differences most likely include gene copy number variations, or even rearrangements of regions between chromosomes. We found indirect evidence for such large rearrangements when we analyzed the genome-wide linkage of markers. Several regions in which seemingly correct haplotypes were in strong linkage disequilibrium with both neighboring regions and regions on other chromosomes were found (data not shown). In addition, error-types do not occur at random. In our dataset, >70% of all errors were caused by two error-types (HBH and AHA) (Figure 7A), indicating a common, systematic cause for both error-types. Future GBS pipelines could address those issues, either by taking into account improved reference genome information, through linkage disequilibrium filtering, or by rearranging marker order. However, at the moment, GBS software that explicitly addresses the problems associated with a wide cross does not exist.

When we established our GBS pipeline, we noticed several irregularities in the genome-wide SNP statistics. For example, we noticed that several regions of the genome were sparsely covered with SNPs (Figure 3). Also, we noted that, in several regions, parental allele frequency deviated from the expected 1:2:1 ratio (Figure 4). It is important to note that this population is affected by reproductive incompatibilities, and we had to routinely use embryo-rescue to propagate plant materials. It is very likely that the deviating allele frequency is a consequence of reproductive incompatibility, which has its genetic basis in these regions. To further analyze this, it would be necessary to genotype the offspring of multiple F1 crosses. We suggest that GBS might be a useful tool to study reproductive isolation and preferential transmission, since it can quickly define regions with allele distortion.

### Conclusion

In summary, we show an application of GBS to perform linkage analysis in a rice F2 population. We also provide an example of how to plan and carry out adequate, cost effective, reduced-representation sequencing. With our dataset, we successfully detected QTL for tiller number on chromosomes 1, 3, 4, and 8, which we could partially confirm using ILs. For future GBS genotyping efforts, we suggest evaluating enzyme choice, multiplexing of libraries and post-processing to meet the requirements of the desired post-GBS analyses.

## Supplementary Material

Supplemental material is available online at www.g3journal.org/lookup/suppl/doi:10.1534/g3.116.038190/-/DC1.

## References

[bib1] Begum H. Spindel J. E. Lalusin A. Borromeo T. Gregorio G., 2015 Genome-wide association mapping for yield and other agronomic traits in an elite breeding population of tropical rice *(Oryza sativa*). PLoS One 10: e0119873. 25785447 10.1371/journal.pone.0119873 PMC4364887

[bib2] Broman K. W. Sen Ś., 2009 *A Guide to QTL Mapping with R/qtl*. Springer, New York, NY.

[bib3] Broman K. W. Wu H. Sen S. Churchill G. A., 2003 R/qtl: QTL mapping in experimental crosses. Bioinformatics 19: 889–890. 12724300 10.1093/bioinformatics/btg112

[bib4] Burrell A. M. Pepper A. E. Hodnett G. Goolsby J. A. Overholt W. A., 2015 Exploring origins, invasion history and genetic diversity of *Imperata cylindrica* (L.) P. Beauv. (Cogongrass) in the United States using genotyping by sequencing. Mol. Ecol. 24: 2177–2193. 25864837 10.1111/mec.13167

[bib5] Danecek P. Auton A. Abecasis G. Albers C. A. Banks E., 2011 The variant call format and VCFtools. Bioinformatics 27: 2156–2158. 21653522 10.1093/bioinformatics/btr330 PMC3137218

[bib6] Darvasi A., 1998 Experimental strategies for the genetic dissection of complex traits in animal models. Nat. Genet. 18: 19–24. 9425894 10.1038/ng0198-19

[bib7] Davey J. W. Hohenlohe P. A. Etter P. D. Boone J. Q. Catchen J. M., 2011 Genome-wide genetic marker discovery and genotyping using next-generation sequencing. Nat. Rev. Genet. 12: 499–510. 21681211 10.1038/nrg3012

[bib8] De Donato M. Peters S. O. Mitchell S. E. Hussain T. Imumorin I. G., 2013 Genotyping-by-sequencing (GBS): a novel, efficient and cost-effective genotyping method for cattle using next-generation sequencing. PLoS One 8: e62137. 23690931 10.1371/journal.pone.0062137 PMC3656875

[bib9] Doyle J. J. Doyle J. L., 1987 A rapid DNA isolation procedure for small quantities of fresh leaf tissue. Phytochem. Bull. 19: 11–15.

[bib10] Duitama J. Silva A. Sanabria Y. Cruz D. F. Quintero C., 2015 Whole genome sequencing of elite rice cultivars as a comprehensive information resource for marker assisted selection. PLoS One 10: e0124617. 25923345 10.1371/journal.pone.0124617 PMC4414565

[bib11] Elmer I. Humira S. François B., 2015 Association mapping of QTLs for sclerotinia stem rot resistance in a collection of soybean plant introductions using a genotyping by sequencing (GBS) approach. BMC Plant Biol. 15: 5. 25595526 10.1186/s12870-014-0408-y PMC4304118

[bib12] Elshire R. J. Glaubitz J. C. Sun Q. Poland J. A. Kawamoto K., 2011 A robust, simple genotyping-by-sequencing (GBS) approach for high diversity species. PLoS One 6: e19379. 21573248 10.1371/journal.pone.0019379 PMC3087801

[bib13] Glaubitz J. C. Casstevens T. M. Lu F. Harriman J. Elshire R. J., 2014 TASSEL-GBS: a high capacity genotyping by sequencing analysis pipeline. PLoS One 9: e90346. 24587335 10.1371/journal.pone.0090346 PMC3938676

[bib14] Gualdrón Duarte J. L. Bates R. O. Ernst C. W. Raney N. E. Cantet R. J., 2013 Genotype imputation accuracy in a F2 pig population using high density and low density SNP panels. BMC Genet. 14: 38. 23651538 10.1186/1471-2156-14-38 PMC3655050

[bib15] Hahne F. Ivanek R., 2016 Visualizing genomic data using Gviz and Bioconductor, pp. 335–351 in Statistical Genomics: Methods and Protocols, edited by Mathé E. Davis S. Springer New York, New York, NY. 10.1007/978-1-4939-3578-9_16 27008022

[bib16] He J. Zhao X. Laroche A. Lu Z.-X. Liu H., 2014 Genotyping-by-sequencing (GBS), an ultimate marker-assisted selection (MAS) tool to accelerate plant breeding. Front. Plant Sci. 5: 484. 25324846 10.3389/fpls.2014.00484 PMC4179701

[bib17] Heffelfinger C. Fragoso C. A. Moreno M. A. Overton J. D. Mottinger J. P., 2014 Flexible and scalable genotyping-by-sequencing strategies for population studies. BMC Genomics 15: 979. 25406744 10.1186/1471-2164-15-979 PMC4253001

[bib18] Honsdorf N. March T. Hecht A. Eglinton J. Pillen K., 2014 Evaluation of juvenile drought stress tolerance and genotyping by sequencing with wild barley introgression lines. Mol. Breed. 34: 1475–1495.

[bib19] Huang Y.-F. Poland J. A. Wight C. P. Jackson E. W. Tinker N. A., 2014 Using genotyping-by-sequencing (GBS) for genomic discovery in cultivated oat. PLoS One 9: e102448. 25047601 10.1371/journal.pone.0102448 PMC4105502

[bib20] Hyma K. E. Barba P. Wang M. Londo J. P. Acharya C. B., 2015 Heterozygous mapping strategy (HetMappS) for high resolution genotyping-by-sequencing markers: a case study in grapevine. PLoS One 10: e0134880. 26244767 10.1371/journal.pone.0134880 PMC4526651

[bib21] Johnson J. L. Wittgenstein H. Mitchell S. E. Hyma K. E. Temnykh S. V., 2015 Genotyping-by-sequencing (GBS) detects genetic structure and confirms behavioral QTL in tame and aggressive foxes (*Vulpes vulpes*). PLoS One 10: e0127013. 26061395 10.1371/journal.pone.0127013 PMC4465646

[bib22] Kawahara Y. de la Bastide M. Hamilton J. P. Kanamori H. McCombie W. R, 2013 Improvement of the *Oryza sativa* Nipponbare reference genome using next generation sequence and optical map data. Rice (N. Y.) 6: 4. 24280374 10.1186/1939-8433-6-4 PMC5395016

[bib23] Kearsey M. J. Farquhar A. G., 1998 QTL analysis in plants; where are we now? Heredity 80: 137–142. 9503632 10.1046/j.1365-2540.1998.00500.x

[bib24] Krishnan S. G. Waters D. L Henry R. J, 2014 Australian wild rice reveals pre-domestication origin of polymorphism deserts in rice genome. PLoS One 9: e98843. 24905808 10.1371/journal.pone.0098843 PMC4048307

[bib25] Li H. Durbin R., 2009 Fast and accurate short read alignment with Burrows-Wheeler transform. Bioinformatics 25: 1754–1760. 19451168 10.1093/bioinformatics/btp324 PMC2705234

[bib26] Lin M. Cai S. Wang S. Liu S. Zhang G., 2015 Genotyping-by-sequencing (GBS) identified SNP tightly linked to QTL for pre-harvest sprouting resistance. Theor. Appl. Genet. 128: 1385–1395. 25851002 10.1007/s00122-015-2513-1

[bib27] Liu H. Bayer M. Druka A. Russell J. R. Hackett C. A., 2014 An evaluation of genotyping by sequencing (GBS) to map the *Breviaristatum-e* (*ari-e*) locus in cultivated barley. BMC Genomics 15: 104. 24498911 10.1186/1471-2164-15-104 PMC3922333

[bib28] Loman N. J. Misra R. V. Dallman T. J. Constantinidou C. Gharbia S. E., 2012 Performance comparison of benchtop high-throughput sequencing platforms. Nat. Biotechnol. 30: 434–439. 22522955 10.1038/nbt.2198

[bib29] Lu F. Lipka A. E. Glaubitz J. Elshire R. Cherney J. H., 2013 Switchgrass genomic diversity, ploidy, and evolution: novel insights from a network-based SNP discovery protocol. PLoS Genet. 9: e1003215. 23349638 10.1371/journal.pgen.1003215 PMC3547862

[bib30] Poland J. A. Rife T. W., 2012 Genotyping-by-sequencing for plant breeding and genetics. Plant Genome 5: 92–102.

[bib31] Poland J. A. Brown P. J. Sorrells M. E. Jannink J.-L., 2012 Development of high-density genetic maps for barley and wheat using a novel two-enzyme genotyping-by-sequencing approach. PLoS One 7: e32253. 22389690 10.1371/journal.pone.0032253 PMC3289635

[bib32] Pootakham W. Jomchai N. Ruang-Areerate P. Shearman J. R. Sonthirod C., 2015 Genome-wide SNP discovery and identification of QTL associated with agronomic traits in oil palm using genotyping-by-sequencing (GBS). Genomics 105: 288–295. 25702931 10.1016/j.ygeno.2015.02.002

[bib33] R Development Core Team, 2008 *R: A Language and Environment for Statistical Computing* R Foundation for Statistical Computing, Vienna, Austria. Available at: http://www.R-project.org.

[bib34] Rabbi I. Y. Hamblin M. T. Kumar P. L. Gedil M. A. Ikpan A. S., 2014 High-resolution mapping of resistance to cassava mosaic geminiviruses in cassava using genotyping-by-sequencing and its implications for breeding. Virus Res. 186: 87–96. 24389096 10.1016/j.virusres.2013.12.028

[bib35] Ramos J. M. Furuta T. Uehara K. Chihiro N. Angeles-Shim R. B., 2016 Development of chromosome segment substitution lines (CSSLs) of *Oryza longistaminata* A. Chev. & Röhr in the background of the elite *japonica* rice cultivar, Taichung 65 and their evaluation for yield traits. Euphytica 210: 151–163.

[bib36] Rice P. Longden I. Bleasby A., 2000 EMBOSS: the European molecular biology open software suite. Trends Genet. 16: 276–277. 10827456 10.1016/s0168-9525(00)02024-2

[bib37] Romay M. C. Millard M. J. Glaubitz J. C. Peiffer J. A. Swarts K. L., 2013 Comprehensive genotyping of the USA national maize inbred seed bank. Genome Biol. 14: R55. 23759205 10.1186/gb-2013-14-6-r55 PMC3707059

[bib38] Rowan B. A. Patel V. Weigel D. Schneeberger K., 2015 Rapid and inexpensive whole-genome genotyping-by-sequencing for crossover localization and fine-scale genetic mapping. G3 5: 385–398. 25585881 10.1534/g3.114.016501 PMC4349092

[bib39] Schröder S. Mamidi S. Lee R. McKain M. R. McClean P. E., 2016 Optimization of genotyping by sequencing (GBS) data in common bean (*Phaseolus vulgaris* L.). Mol. Breed. 36: 1–9.

[bib40] Sen Ś. Churchill G. A., 2001 A statistical framework for quantitative trait mapping. Genetics 159: 371–387. 11560912 10.1093/genetics/159.1.371 PMC1461799

[bib41] Sonah H. O’Donoughue L. Cober E. Rajcan I. Belzile F., 2015 Identification of loci governing eight agronomic traits using a GBS-GWAS approach and validation by QTL mapping in soya bean. Plant Biotechnol. J. 13: 211–221. 25213593 10.1111/pbi.12249

[bib42] Spindel J. Wright M. Chen C. Cobb J. Gage J., 2013 Bridging the genotyping gap: using genotyping by sequencing (GBS) to add high-density SNP markers and new value to traditional bi-parental mapping and breeding populations. Theor. Appl. Genet. 126: 2699–2716. 23918062 10.1007/s00122-013-2166-x

[bib43] Swarts, K., H. Li, J. A. Romero Navarro, D. An, M. C. Romay *et al.*, 2014 Novel methods to optimize genotypic imputation for low-coverage, next-generation sequence data in crop plants. Plant Genome 7. Available at: https://dl.sciencesocieties.org/publications/tpg/abstracts/7/3/plantgenome2014.05.0023.

[bib44] Takagi H. Abe A. Yoshida K. Kosugi S. Natsume S., 2013 QTL-seq: rapid mapping of quantitative trait loci in rice by whole genome resequencing of DNA from two bulked populations. Plant J. 74: 174–183. 23289725 10.1111/tpj.12105

[bib45] Wang L. Hao L. Li X. Hu S. Ge S., 2009 SNP deserts of Asian cultivated rice: genomic regions under domestication. J. Evol. Biol. 22: 751–761. 19243488 10.1111/j.1420-9101.2009.01698.x

[bib46] Wang M. Yu Y. Haberer G. Marri P. R. Fan C., 2014 The genome sequence of African rice (*Oryza glaberrima*) and evidence for independent domestication. Nat. Genet. 46: 982–988. 25064006 10.1038/ng.3044 PMC7036042

[bib47] Zhang Y. Zhang S. Liu H. Fu B. Li L., 2015 Genome and comparative transcriptomics of African wild rice *Oryza longistaminata* provide insights into molecular mechanism of rhizomatousness and self-incompatibility. Mol. Plant 8: 1683–1686. 26358679 10.1016/j.molp.2015.08.006

