# Supplementary figures and images for: Adapting Genotyping-by-Sequencing for Rice F2 Populations
Source: G3 (Bethesda). 2017 Jan 11;7(3):881–93. doi: 10.1534/g3.116.038190 (PMC5345719; doi:10.1534/g3.116.038190)

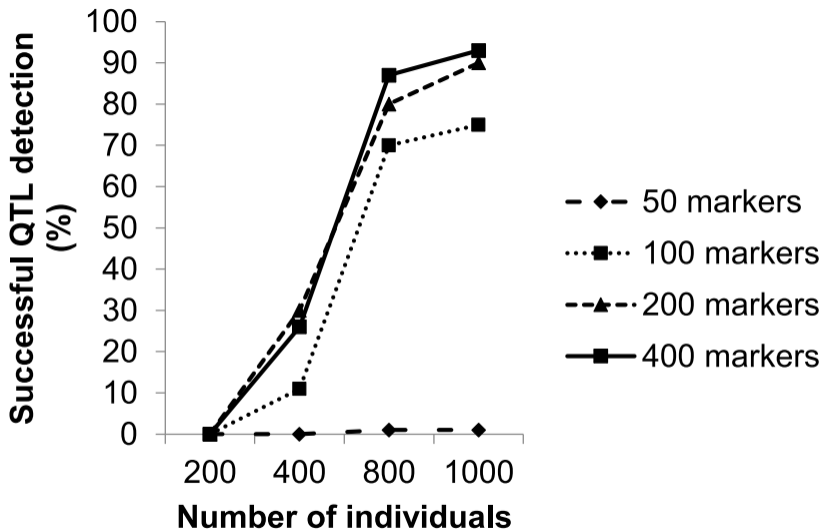

Supplement: Supplementary file 1 [file 881FigureS1.pdf]

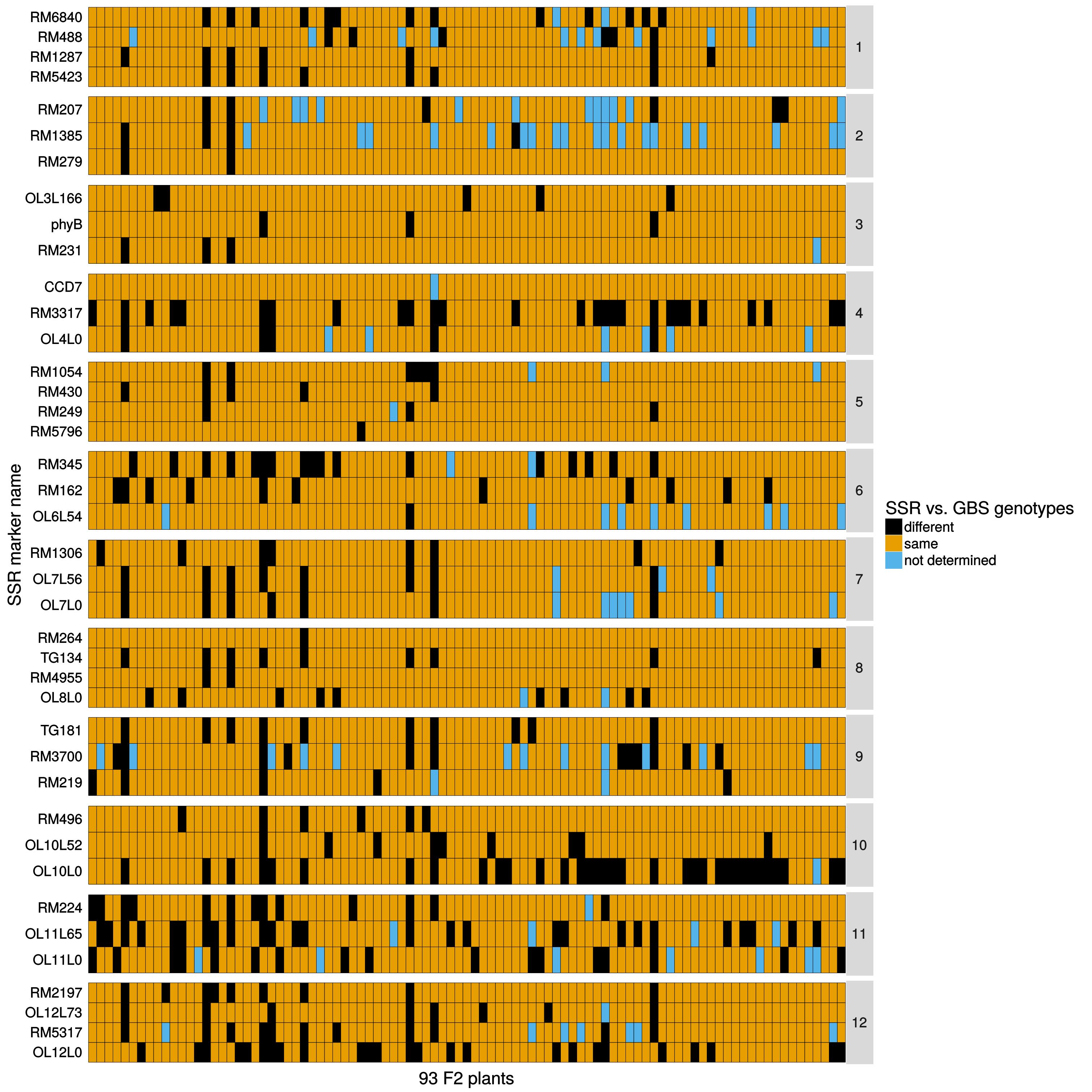

Supplement: Supplementary file 3 [file 881FigureS3.pdf]

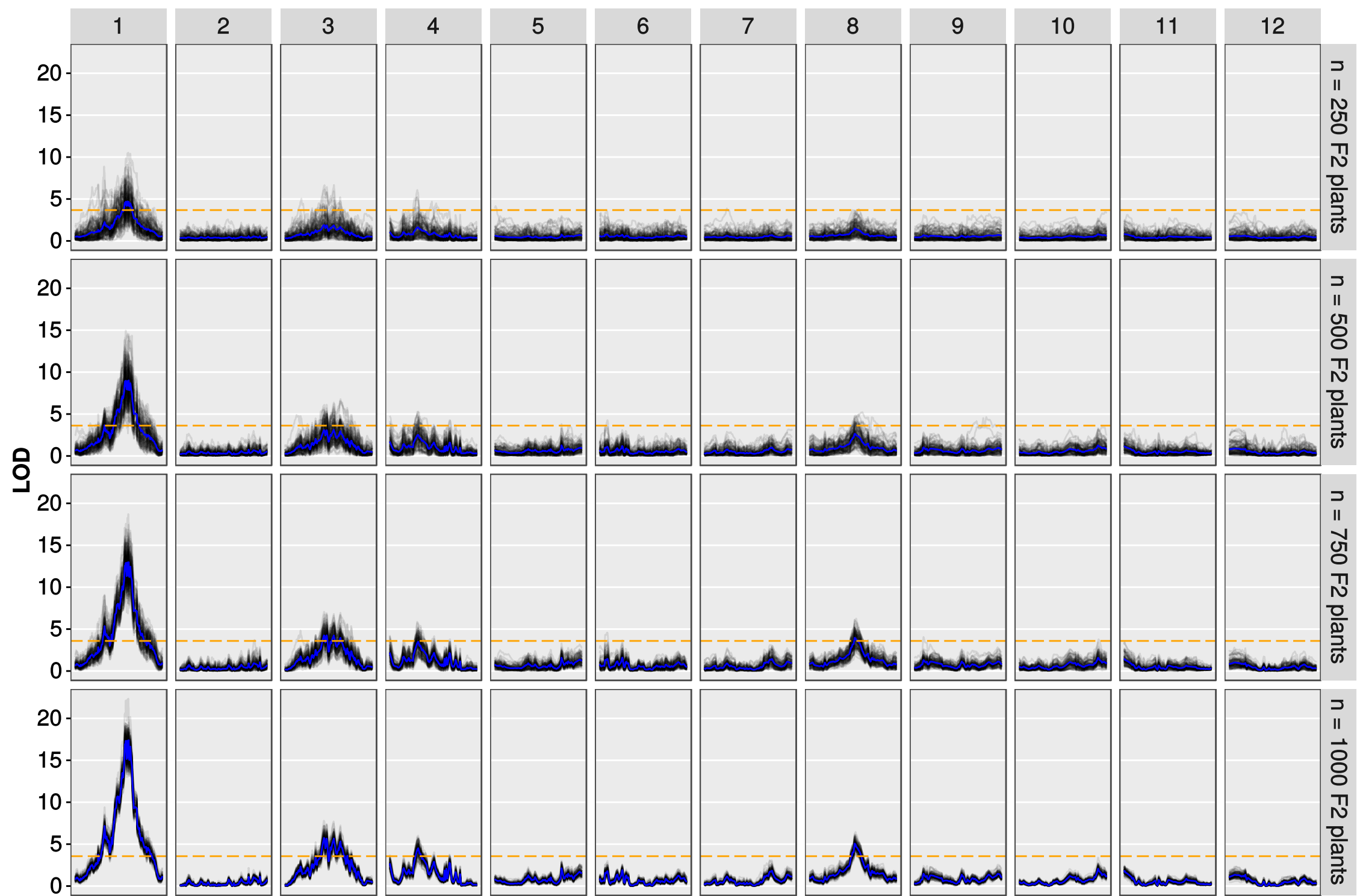

Supplement: Supplementary file 4 [file 881FigureS4.pdf]

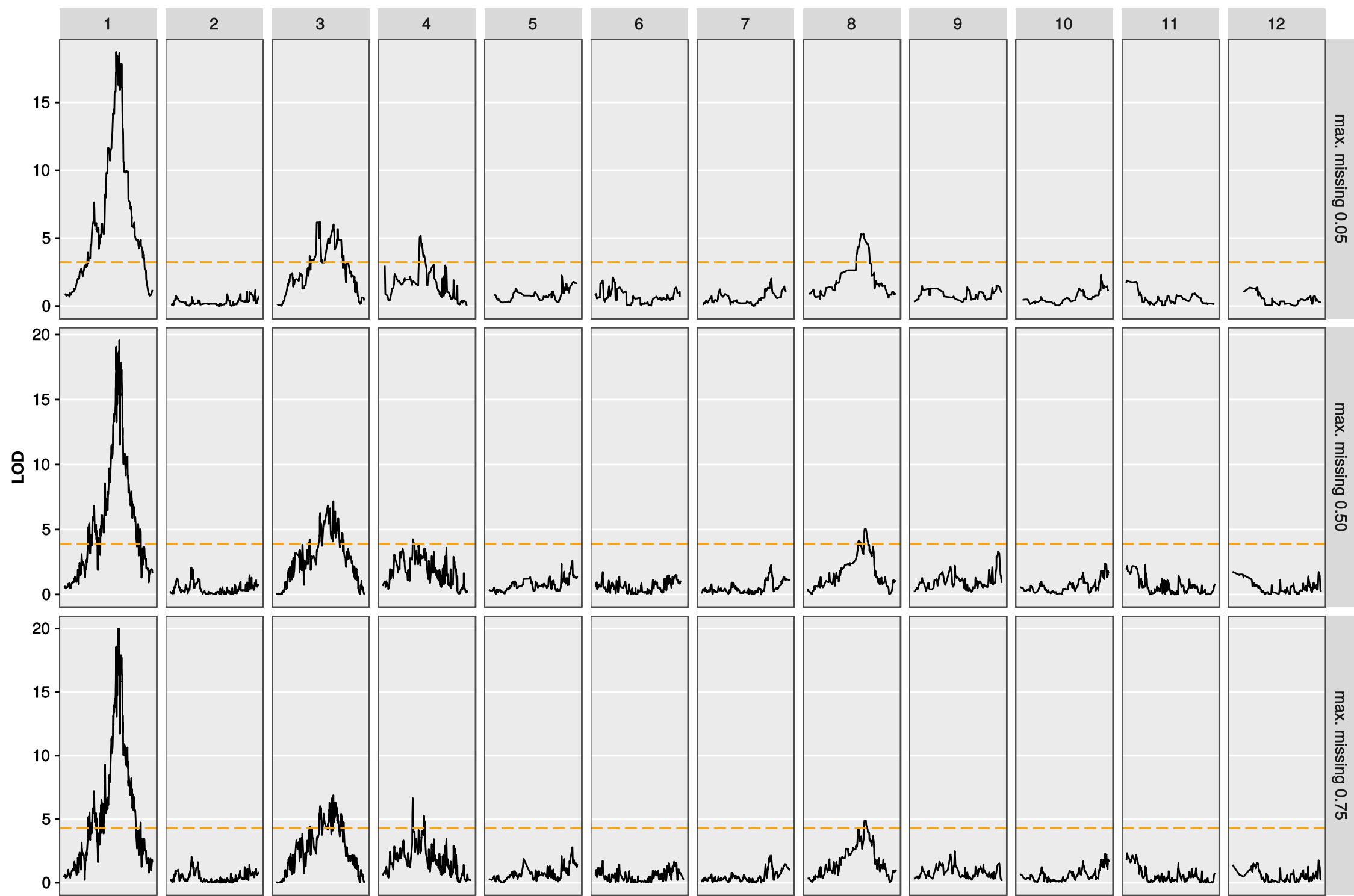

Supplement: Supplementary file 5 [file 881FigureS5.pdf]
