# Supplementary material for: Adapting Genotyping-by-Sequencing for Rice F2 Populations
Source: G3 (Bethesda). 2017 Jan 11;7(3):881–93. doi: 10.1534/g3.116.038190 (PMC5345719; doi:10.1534/g3.116.038190)
Supplement: Supplementary file 2 [file 881FigureS2.pdf]

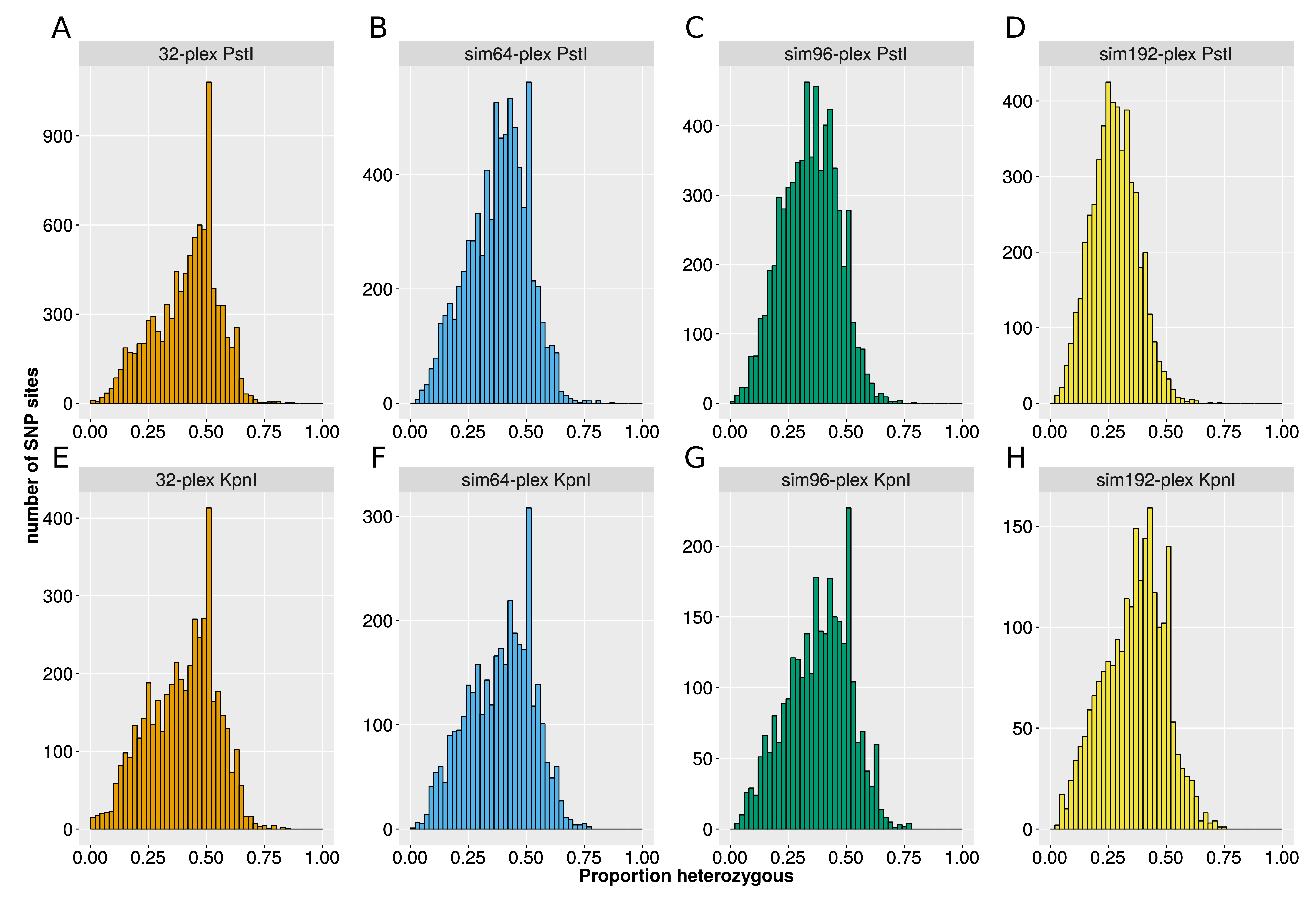

A
32-plex PstI
B
sim64-plex PstI
C
sim96-plex PstI
D
sim192-plex PstI
E
32-plex KpnI
F
sim64-plex KpnI
G
sim96-plex KpnI
H
sim192-plex KpnI
number of SNP sites
Proportion heterozygous
0.00 0.25 0.50 0.75 1.00
