# Supplementary material for: Adapting Genotyping-by-Sequencing for Rice F2 Populations
Source: G3 (Bethesda). 2017 Jan 11;7(3):881–93. doi: 10.1534/g3.116.038190 (PMC5345719; doi:10.1534/g3.116.038190)
Supplement: Supplementary file 10 [file 881TableS3.docx]

Table S3. Number of error-types for each F2 individual and each SNP marker. (.xlsx, 64 KB)

[http://www.g3journal.org/lookup/suppl/doi:10.1534/g3.116.038190/-/DC1/TableS3.xlsx](http://www.g3journal.org/lookup/suppl/doi:10.1534/g3.116.038190/-/DC1/TableS2.xlsx)
