# Supplementary material for: Adapting Genotyping-by-Sequencing for Rice F2 Populations
Source: G3 (Bethesda). 2017 Jan 11;7(3):881–93. doi: 10.1534/g3.116.038190 (PMC5345719; doi:10.1534/g3.116.038190)
Supplement: Supplementary file 15 [file 881FileS1.docx]

File S1. All code necessary to replicate the GBS-pipeline. (.zip, 20 KB)

<http://www.g3journal.org/lookup/suppl/doi:10.1534/g3.116.038190/-/DC1/FileS1.zip>
