# Supplementary material for: Adapting Genotyping-by-Sequencing for Rice F2 Populations
Source: G3 (Bethesda). 2017 Jan 11;7(3):881–93. doi: 10.1534/g3.116.038190 (PMC5345719; doi:10.1534/g3.116.038190)
Supplement: Supplementary file 18 [file 881FileS3.docx]

File S3. Comparison of GBS-derived genotypes and Sanger sequencing-derived genotypes. (.xlsx, 20 KB)

<http://www.g3journal.org/lookup/suppl/doi:10.1534/g3.116.038190/-/DC1/DatasetS3.xlsx>
